# Parieto-frontal mechanisms underlying observation of complex hand-object manipulation

**DOI:** 10.1038/s41598-018-36640-5

**Published:** 2019-01-23

**Authors:** Antonino Errante, Leonardo Fogassi

**Affiliations:** Department of Medicine and Surgery, University of Parma, Parma, 43125 Italy

## Abstract

The observation of actions performed by others is believed to activate the Action Observation Network (AON). Previous evidence suggests that subjects with a specific motor skill show increased activation of the AON during observation of the same skill. The question arises regarding which modulation of the AON occurs during observation of novel complex manipulative actions that are beyond the personal motor repertoire. To address this issue, we carried out a functional MRI study in which healthy volunteers without specific hand motor skills observed videos displaying hand-object manipulation executed by an expert with high manual dexterity, by an actor with intermediate ability or by a naïve subject. The results showed that the observation of actions performed by a naïve model produced stronger activation in a dorso-medial parieto-premotor circuit including the superior parietal lobule and dorsal premotor cortex, compared to observation of an expert actor. Functional connectivity analysis comparing the observation of the naïve model with that of the expert model, revealed increased connectivity between dorsal areas of the AON. This suggests a possible distinction between ventral and dorsal brain circuits involved in the processing of different aspects of action perception, such as kinematics and final action goal.

## Introduction

It is a well-accepted notion in neurophysiology that the observation of actions performed by others activates in the perceiver the same neural structures responsible for the actual execution of the same actions^1^. This mechanism is provided by the mirror neuron system (MNS), originally demonstrated in the monkey, formed by visuomotor neurons that fire both when individuals perform a goal-directed action and when they observe the same, or a similar action, performed by another individual^2–4^.

A similar pattern of cortical activation is present in humans, as demonstrated by several electrophysiological and neuroimaging studies^5–13^. Indeed, the inferotemporal cortex, which constitutes the source of visual input about biological actions to the MNS, activates only during action observation, whereas the inferior parietal and premotor cortices, as well the inferior frontal gyrus (IFG), activate during both action observation and execution^14,15^. However, it has been reported that, in addition to these main nodes, action observation often activates other areas belonging to the so-called Action Observation Network (AON), which includes the superior parietal and dorsal premotor areas^16–18^.

Several studies^19–22^ have indicated that AON plays a strong role in representing previously acquired motor skills, showing that during action observation, the observer’s brain tends to activate more strongly for actions already embodied in the own motor repertoire. The results of most studies on expertise are in agreement with this evidence; such studies have investigated how acquired skilled actions, such as sports or dance moves, affect AON activity while a subject observe movements typical of these actions^20,21,23,24^. These studies, which have typically compared the differential BOLD response in experts and novices, have demonstrated that during observation of the skilled action, AON areas activate more strongly in experts. It is likely that this higher activation reflects the high plasticity of the motor system.

Studies on the relation between AON activity and motor skills have produced little knowledge about brain activation during observation of novel complex hand manipulative actions that go beyond the personal motor repertoire (e.g., actions performed by an actor with a high level of manual dexterity). The first aim of the present fMRI study was to investigate the possible modulation of AON activity caused by the observation of different levels of hand/fingers motor dexterity in naïve participants. The underlying hypothesis is that the AON should be more active when the observed hand actions correspond to the own motor repertoire, and that it should be less active during the processing of hand actions that are beyond the own motor repertoire, such as those performed by an expert model. The second aim was to investigate the modulation induced by the observation of a naïve model versus an expert model on the functional connectivity pattern between brain areas involved in the processing of observed complex object-related manipulative actions.

## Results

### Brain representation of the observation of complex hand actions

**Figure 1:**
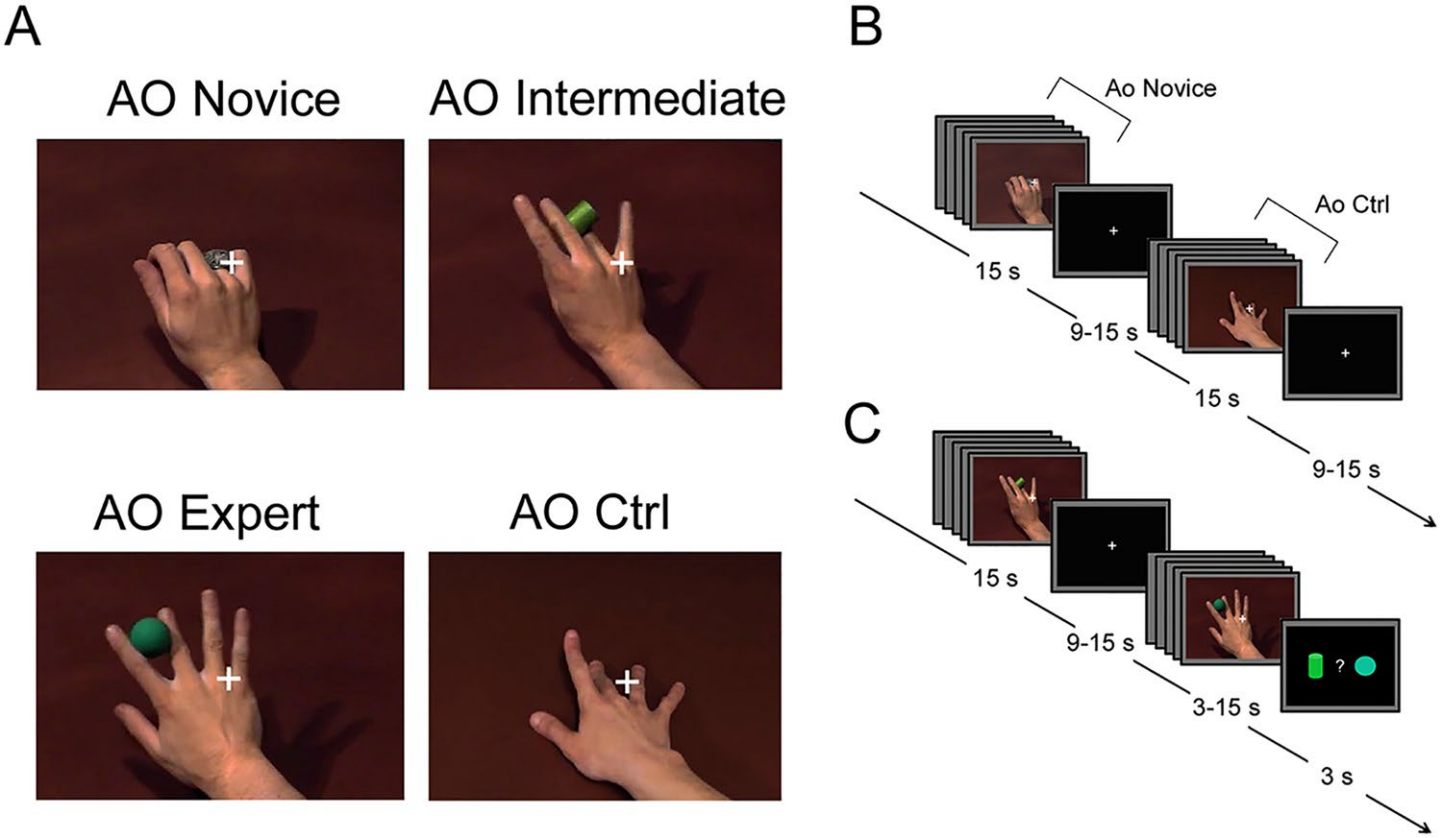
Experimental stimuli and task design. (**A**) Static frames taken from the videos showing actions performed by a model without particular hand skills (AO Novice), by a model with intermediate level of hand ability after 2-months training (AO Intermediate), by an expert model with high level of manual dexterity (AO Expert). In the control condition, participants observed simple extension-flexion fingers movements (AO Ctrl). All movements are shown in a first-person perspective. (**B**) Action observation paradigm, presented in two functional runs, made by independent blocks of 15 s, consisting of five randomly presented videos of the same condition. Each block was interleaved with a *rest* period (9–15 s). (**C**) Example of a Catch-Trial presented at the end of a block (25% of the total number of blocks), in which participants had to indicate the shape of the previously presented object.

Eighteen right-handed humans performed an action observation task in two functional runs after a short training session. They were instructed to passively observe video clips displaying a complex object manipulation performed with the right hand, lasting 3-s each (see Fig. 1A; Supplementary Videos 1–6). There were three experimental conditions: (1) actions performed by an expert actor (professional juggler) with a high level of hand/finger dexterity (action observation Expert, *AO Expert*); (2) actions performed by an actor with an intermediate level of manual dexterity (action observation intermediate, *AO Intermediate*) after two-months of training for improving his manipulation skills, consisting in object manipulation practiced for 1 h every day; and (3) actions performed by a naïve subject, without any particular experience in object manipulation or manual skills (action observation novice, *AO Novice*). In addition to the video clips showing object manipulation, 3-s video clips showing sequences of random finger movements performed with the index, middle, ring or little finger (right hand) were used in order to control for the mere general processing of biological motion. For this condition, we used four random sequences of extension-flexion finger movements (action observation Control, *AO Ctrl*). In 25% of the blocks, participants had to provide an explicit response (catch-trials), using a response-pad placed inside the scanner, concerning the shape of the object observed in the last video clip, thus unrelated to motor aspects of the task (see Methods for details). Mean response accuracy recorded during catch-trials was 94.4% (SD = 10.6%) for trials related to AO Novice, 97.2% (SD = 8%) for trials related to AO Intermediate and 95.8% (SD = 9.5%) for trials related to AO Expert. No statistical differences (significance level set at *P* < 0.01) between the number of correct answers for AO Novice, Intermediate and Expert (*F*_2,17_ = 0.32; *P* = 0.72) were observed.

**Figure 2:**
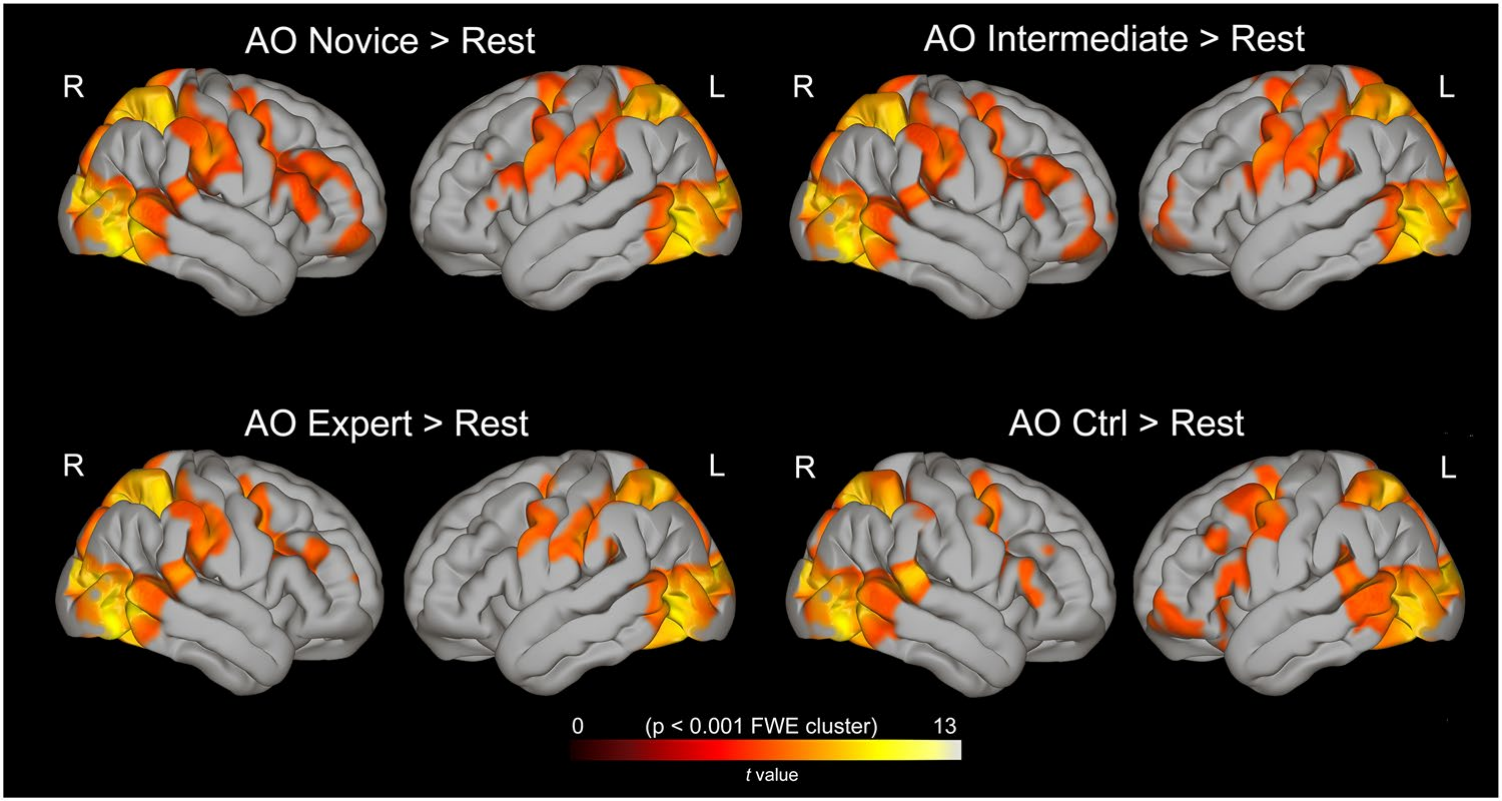
Brain activations during the experimental and control conditions. The activations, reported on both hemispheres, result from the contrast between each experimental condition and rest. All activations are rendered into a standard MNI brain template (*P* < 0.001 FWE corrected at cluster level). L, left hemisphere; R, right hemisphere.

The observation of complex hand-object manipulation (*AO Novice*, *AO Intermediate*, *AO Expert*) produced common activations in areas belonging to the parieto-premotor AON (Fig. 2). Figure 2 shows averaged multi-subject activations displayed on a cortical high-resolution Montreal Neurological Institute (MNI) template available on Conn Toolbox for SPM12. The maps were generated using the contrast images *AO Novice* vs *rest*, *AO Intermediate* vs *rest* and *AO Expert* vs *rest* (see Methods). All the activations revealed in the group analysis were analysed using a statistical threshold of *P* < 0.001, FWE corrected at cluster level. The activation pattern was largely symmetrical, although some clusters were present only in one hemisphere. Common activations included clusters in the occipito-temporal, parietal and premotor cortices. The occipito-temporal activation reached its maximum near the posterior part of the inferior temporal sulcus (ITS) and the posterior middle temporal gyrus (pMTG). This activation showed two branches, one extending dorsally to the posterior end of the superior temporal sulcus (pSTS), and a ventral branch extending into the occipito-temporal sulcus (OTS). The parietal activation included the inferior (IPL) and the superior (SPL) parietal lobules, as well as the ventral, dorsal and anterior intraparietal sulcus (vIPS, dIPS, aIPS). In the frontal lobe, common activations were found in the dorsal and ventral premotor cortex (PMd/PMv), as well as the inferior frontal gyrus (IFG, BA44) and the dorsolateral prefrontal cortex (DLPFC) in the right hemisphere. Subcortical activation included the thalamus, putamen and caudate nucleus, bilaterally.

The activation clusters resulting from the observation of control movements (*AO Ctrl vs rest*), consisting in simple finger movements without objects, revealed a less extended symmetrical pattern (total number of voxels: LH = 5545, RH = 5693) compared to AO Novice (total number of voxels: LH = 5921, RH = 6102) and AO Intermediate (total number of voxels: LH = 5863, RH = 5813). In particular, activated clusters included the bilateral occipito-temporal cortex (STS, MTG, ITG), the posterior parietal cortex (SPL, IPS) and, in the frontal lobe, an area at the border between the PMv and PMd.

Interestingly, because the total time exposure to videos with different durations may have been a confound, we modelled this factor in the analysis. The results of this control analysis revealed no activations in the parietal and premotor areas of the AON associated with different stimulus durations. Only voxels within the visual area hOc4v (V4v) bilaterally were activated by temporal differences to stimuli exposure (see Suppl. Fig. S1).

### Observation of actions performed with different levels of expertise versus control

**Figure 3:**
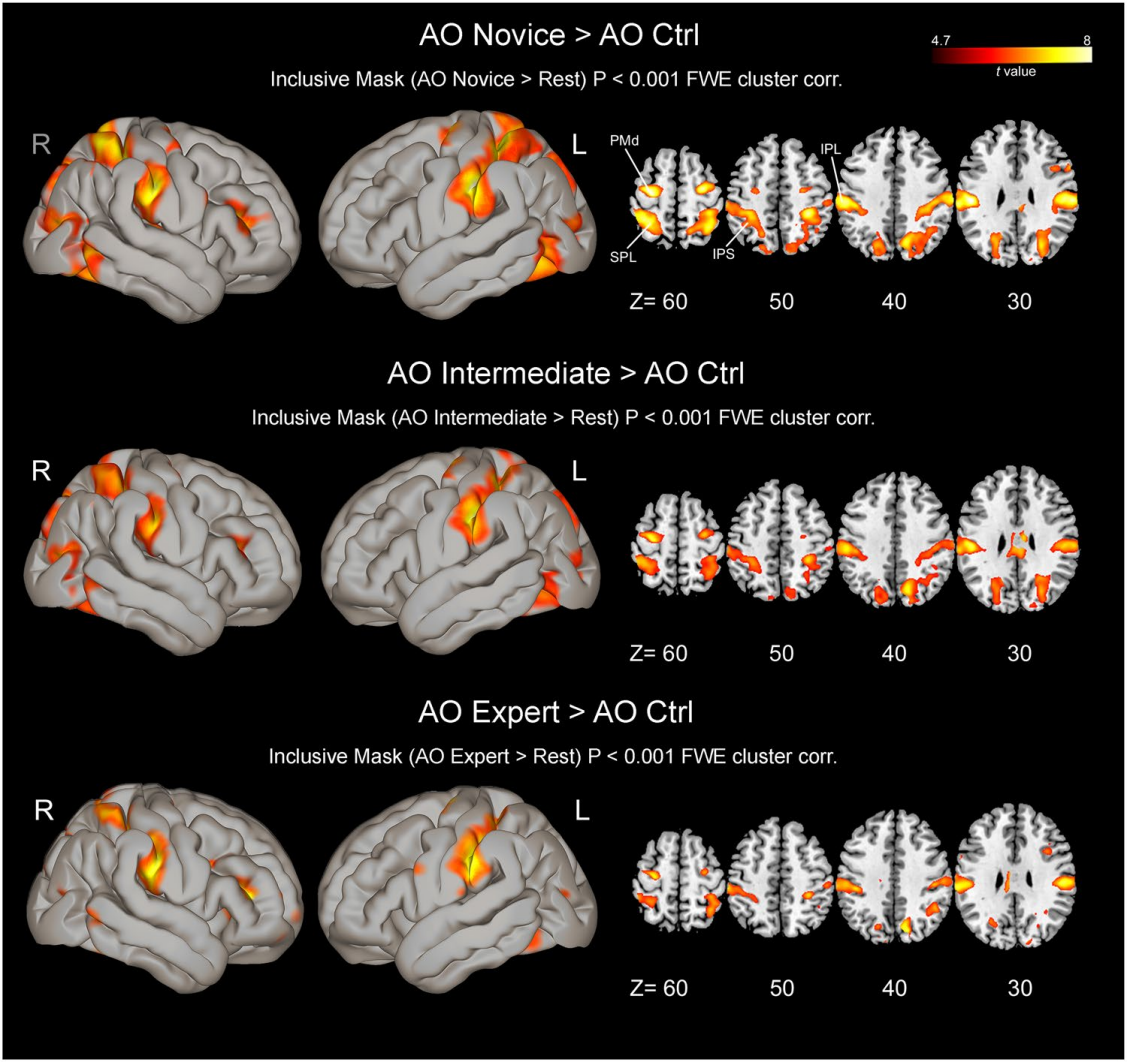
Brain activations resulting from direct contrasts between experimental and control conditions during the action observation task, masked using an inclusive contrast image derived from the corresponding contrast vs rest (*AO Novice* vs rest, *AO Intermediate* vs rest and *AO Expert* vs rest). All activations are rendered into a standard MNI brain template (*P* < 0.001, FWE corrected at cluster level) and in four representative axial slices. IPL = inferior parietal lobule; IPS = intraparietal sulcus; PMd = dorsal premotor cortex; SPL = superior parietal lobule. Other conventions as in Fig. 2.

Compared to the control condition, the observation of complex hand-object manipulations performed by a naïve actor without particular manipulation skills resulted in a strong activation of the AON, including the ITG, IPL, supramarginal gyrus (SMG), IPS, SPL, PMd cortex and IFG, as well as the ventral prefrontal cortex in the right hemisphere (Fig. 3; Supplementary Table S1; *P* < 0.001, FWE corrected at cluster level). Other regions showing consistent activation were the lobule VI of the cerebellum, bilaterally, and the cingulate cortex.

The comparison between the observation of complex manipulative actions performed by an actor with intermediate hand manipulation skill versus control observation showed clusters of activation localized in regions common to the observation of novice, including the IPL/SMG and IPS, belonging to the parietal region, and the PMd cortex in the frontal lobe, as well as the IFG exclusively in the right hemisphere. Other regions activated were the MTG/ITG, cingulate cortex and cerebellum.

The comparison between observation of hand-object manipulative actions performed by the expert actor with high hand skills versus control observation revealed a less extended activation within the AON regions compared to the other experimental conditions. Regions consistently activated were the PMd cortex bilaterally and the IFG in both hemispheres, revealing a stronger response of this region during the observation of skilled actions. In the parietal region, activation clusters were found in the IPL/SMG and the IPS bilaterally. However, the SPL region was more strongly activated in the right hemisphere, than in the left one.

**Figure 4:**
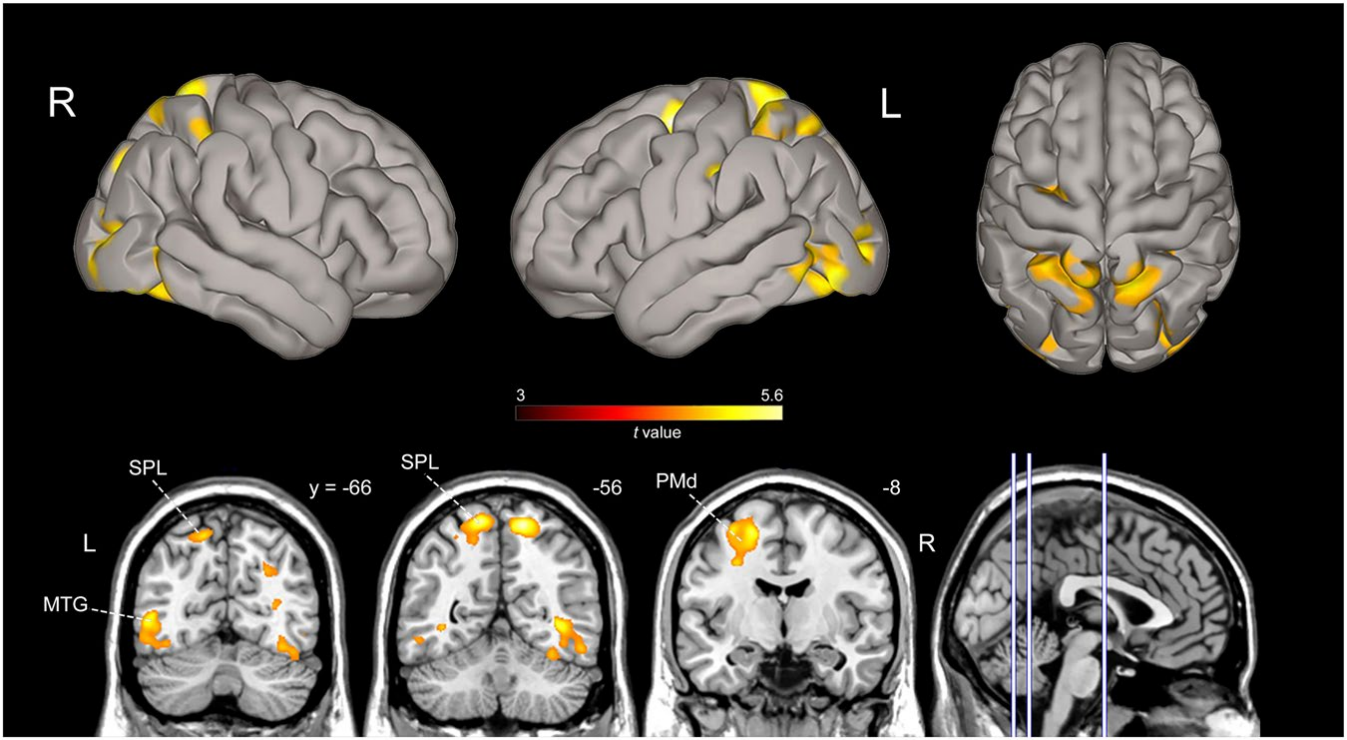
Brain activation map obtained by the contrast between observation of actions performed by novice model and those performed by the expert one (*AO Novice* vs *AO Expert*). All activations are rendered into a standard MNI brain template (*P* < 0.001, FWE corrected at cluster level). Vertical lines on the parasagittal view indicate approximate locations of the three displayed different coronal sections. Abbreviations: MTG, middle temporal gyrus. Other conventions as in Figs 2 and 3.

Finally, we directly contrasted brain activation induced by novice vs expert model observation (Fig. 4). The comparison revealed a parieto-frontal dorsal network of areas mostly lateralized to the left hemisphere including the SPL and IPS within the parietal region, the PMd cortex and other clusters of activation in the ITG bilaterally (for MNI coordinates of activations local maxima see Table 1). The inverse contrast and the other possible comparisons did not show any further differences between conditions.

**Table 1:** Brain areas showing differential activation for the main effect of type of observed model (AO Novice vs AO Expert).

Anatomical region	Left Hemisphere				Right Hemisphere			
	x	y	z	Z-score	x	y	z	Z-score
**AO Novice vs AO Expert**								
Superior Parietal Lobule	−14	−56	66	4.81	16	−54	64	5.28
Precentral Gyrus	−32	−8	60	3.98				
Inferior Parietal Lobule	−50	−20	32	4.34				
Intraparietal Sulcus	−32	−50	50	3.17	34	−40	44	3.96
Superior Frontal Gyrus	−22	−10	52	3.93				
Inferior Occipital Gyrus	−22	−86	−4	4.37	30	−85	−2	4.66
Thalamus					26	−26	−2	4.57
Fusiform Gyrus	−37	−70	−14	4.38				
Middle Temporal Gyrus	−50	−66	−2	4.23				

Only regions surviving a threshold of *P* < 0.001, FWE-corrected at cluster level are reported. Coordinates correspond to local maxima of the respective clusters and are defined in MNI standard space.

### Action execution Localizer

In order to restrict our analysis to the main areas of the AON/MNS, we identified regions activated during motor execution. Previous studies see^25^ have established that manipulating an object involves both motor and sensory components, and we therefore expected that several brain areas subserving this function would be activated. During action execution, participants were instructed to perform an object manipulation with their right hand. The brain activations were observed both at a multisubject level (*P* < 0.05, FWE corrected at voxel level) and in single-subject maps (*P* < 0.001, FWE corrected at cluster level). These brain areas included the primary motor cortex (M1), primary somatosensory cortex (S1), PMd and PMv cortices, IFG, supplementary motor area (SMA), cingulate cortex, IPL/SPL and IPS (see Suppl. Fig. S2). Most activations were lateralized or stronger in the left hemisphere compared to the right, as was expected for normal right-handed participants moving their right hand. Primary motor and primary somatosensory cortex activation was strongly lateralized to the left hemisphere. We used multisubject maps to localize AON areas involved also in execution of complex object manipulation in order to extract the BOLD time-course related to action observation conditions. At single-subject level, we also entered the Action Execution Localizer as inclusive mask in the conjunction analysis between Action Observation contrasts in order to define the SPL, IPS, PMd and PMv seed regions for the subsequent ROI analyses and functional connectivity analyses.

### ROI Analysis

**Figure 5:**
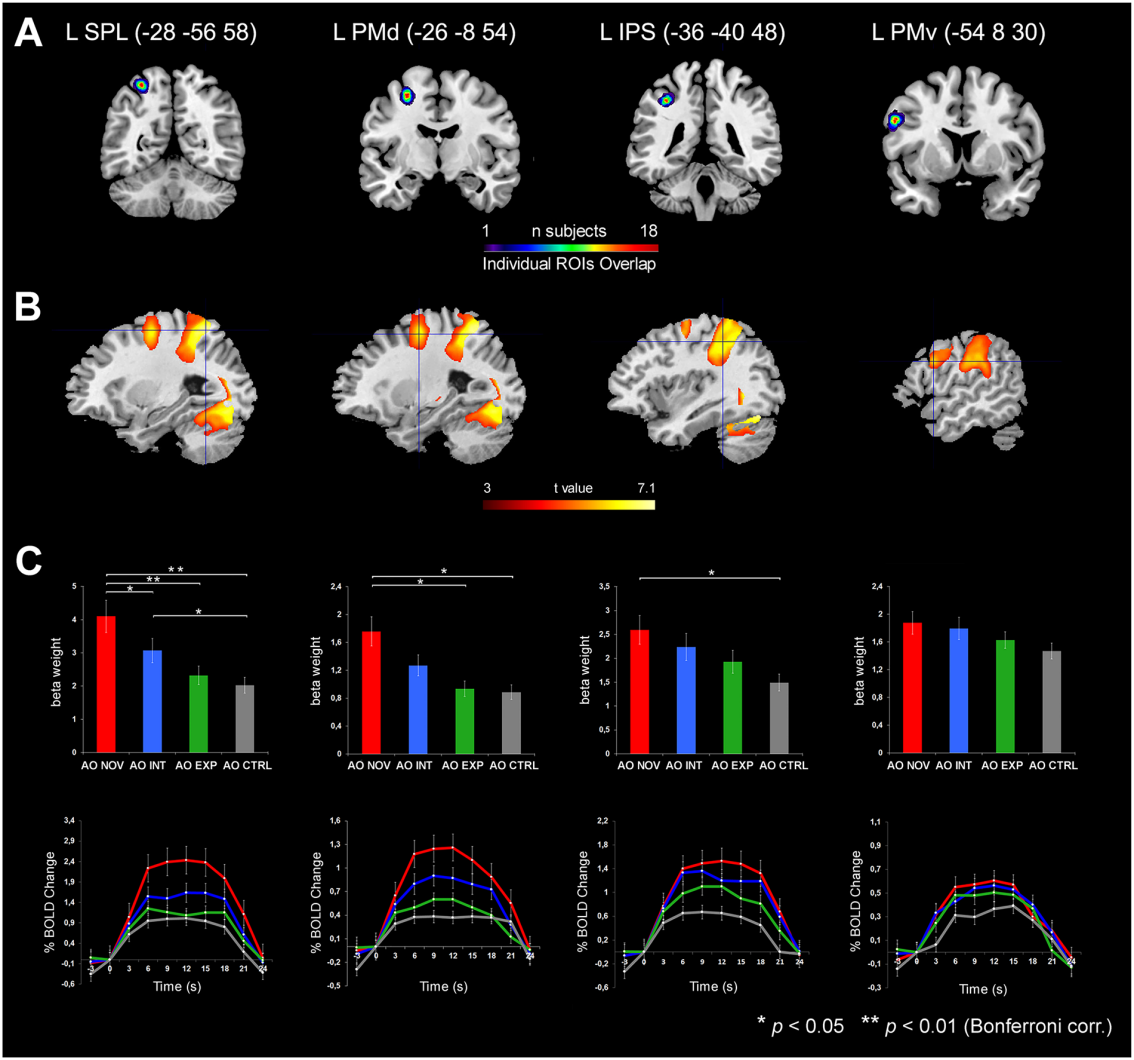
Results of the ROI analysis. (**A**) Coronal sections showing the overlap maps of individual SPL, PMd, IPS and PMv ROIs (radius 4 mm). For each region, the mean coordinates of maximum overlap are reported on the bottom of the section (radius 4 mm ROIs ± 4 mm). (**B**) Parasagittal sections showing the activations resulting from the conjunction between *AO Novice* AND *AO Intermediate* AND *AO Expert*, masked with an inclusive contrast image corresponding to action execution Localizer activations (*P* < 0.05). Crosshair indicate the centre of the overlap for each ROI. **C)** *Top*, histograms showing the averaged magnitude of activation (parameter estimate) in each ROI. Asterisk indicates significant differences set at *P* < 0.05 (*) and *P* < 0.01 (**), corrected using Bonferroni test for multiple comparison. *Bottom*, line graphs indicating the time-course response across peri-stimulus time in left ROIs for all experimental and control conditions. In both histograms and line graphs, vertical lines indicate SEM.

The most important comparison was that performed in order to evaluate a possible differential response of the AON in relation to the expertise of the model observed in the videos. To do this, a ROI analysis has been performed in four parieto-frontal areas activated, considered involved both in action observation and execution (left SPL, left PMd, left IPS and left PMv; see Fig. 5A,B). The identified ROIs are in agreement with previous metanalysis on action observation in humans^14,15^ and previous studies showing overlap in activations during action observation and execution^10,13^. The four ROIs were defined in subject’s native space, before normalization, and then mapped into the standard MNI space (see Methods). We used a conjunction analysis between GLM main contrasts, masked using an inclusive contrast image corresponding to motor execution (*Act_Exe* vs *rest*). The averaged hemodynamic response (histograms of Fig. 5C) has been analysed at group level using a 4 × 4 repeated measures analysis of variance (rmANOVA) with *Condition* (AO Novice, AO Intermediate, AO Expert, AO Ctrl) and *ROI* (SPL, PMd, IPS, PMv) as repeated measure factors. Post-hoc comparisons were computed by using paired-sample *t*-tests with Bonferroni correction for multiple comparisons (alpha set to *P* < 0.003). The ROI analysis revealed a significant main effect for *Condition* [*F* (3, 54) = 15.35, *P* < 0.001, *η*^2^ = 0.46], with higher response for observation of novice than expert (*P* < 0.0001) and control (*P* < 0.0001), and observation of intermediate than control observation (*P* < 0.003). We found also a significant main effect for *ROI* [*F* (3, 54) = 20.64, *P* < 0.001, *η*^2^ = 0.53] with higher BOLD response in SPL region compared to PMd (*P* < 0.0001), IPS (*P* < 0.003) and PMv (*P* < 0.0001). Moreover, BOLD signal in PMd ROI was higher than in IPS ROI (*P* < 0.003). The interaction between *Condition* × *ROI* was also significant [*F* (9, 162) = 3.84, *P* < 0.001, *η*^2^ = 0.17]. Post-hoc comparisons showed that left SPL (Fig. 5C) was more active following the observation of object manipulation performed by the novice actor, compared to that executed with an intermediate level of expertise (*P* < 0.003), high level of expertise (*P* < 0.0001), and observation control (*P* < 0.0001). Similar results were obtained when exploring the hemodynamic response in PMd cortex during the four experimental conditions. However, in this case, we found a different incremental effect, with stronger response during the observation of novice model compared to expert (*P* < 0.003), but not compared to intermediate one. Furthermore, similarly to SPL, even in PMd cortex BOLD change was greater for the observation of novice model compared to control observation (*P* < 0.003). A significant effect was found also in IPS ROI, showing a stronger BOLD response for actions performed by novice compared to control observation (*P* < 0.003). However, in the IPS region no difference was found between activations between novice vs intermediate or expert, and intermediate vs expert conditions. Finally, in PMv there was no difference between BOLD activations in the four experimental conditions. Note, however, that there is a tendency, although not significant, for a different response between novice vs expert condition.

An additional ROI analysis was performed also in visual areas V1 and MT/V5 in order to control the effect of low-level visual features. However, there were no differences in BOLD response for different observation conditions in these early or associative visual areas (ROI1, Left V1 [*F*_3,17_ = 1.22, *P* = 0.18]; ROI2, Right V1 [*F*_3,17_ = 0.73, *P* = 0.52]; ROI3, Left MT/V5 [*F*_3,17_ = 1.36, *P* = 0.12;] ROI4, Right MT/V5 [*F*_3,17_ = 0.89, *P* = 0.65]) (see Suppl. Fig. S3).

### Regression analysis

**Figure 6:**
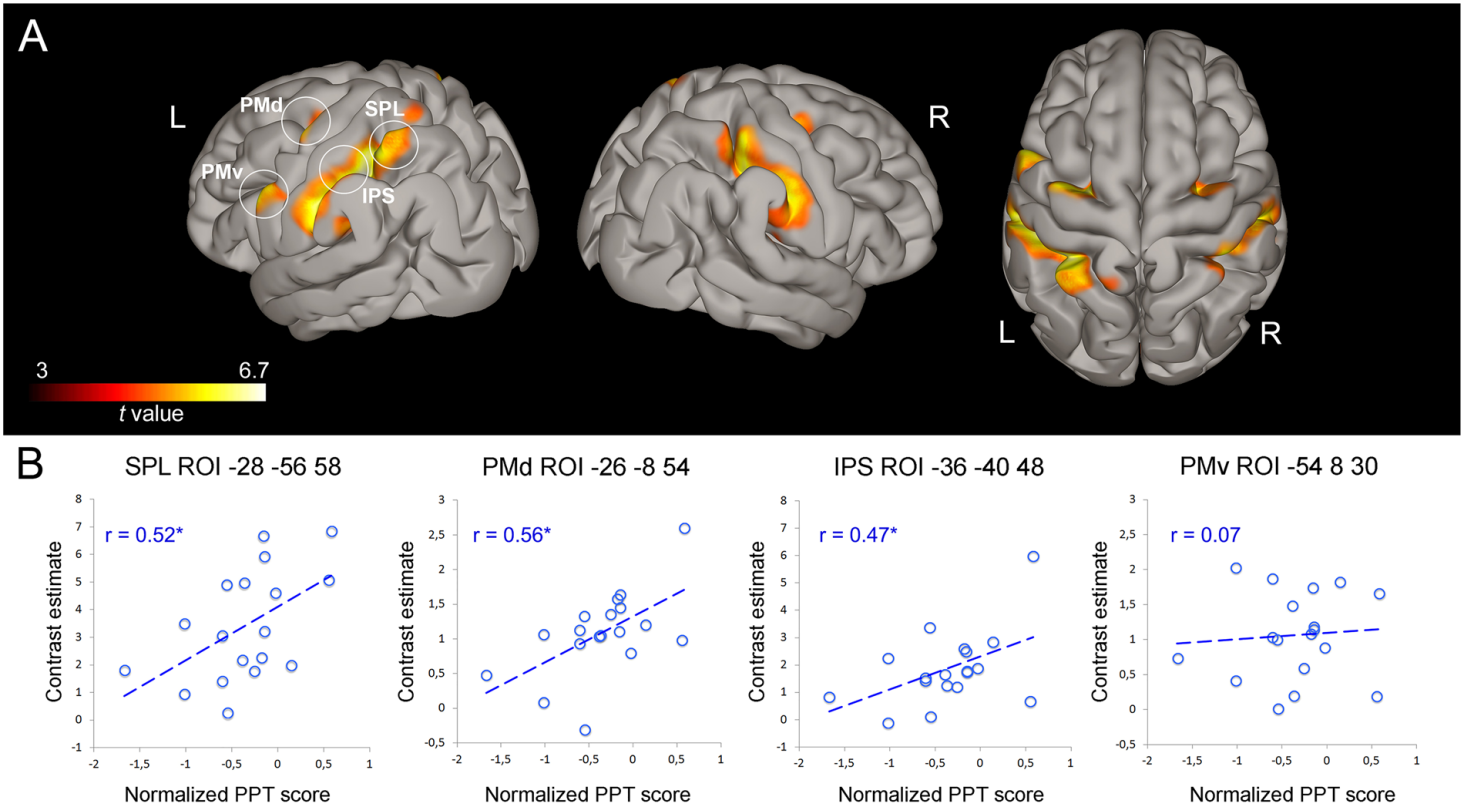
Results of the Regression analysis. (**A**) Activation maps resulting from the conjunction analysis between AO Expert and Act Exe Localizer, rendered into a MNI standard template (*P* < 0.001, FWE corrected at cluster-level). (**B**) Scatterplots showing the correlation between BOLD signal change during the AO Expert condition and the normalized PPT individual scores obtained by participants using the right hand (hand ability score). On the top of each plot the MNI coordinates corresponding to the mean centre of individual ROIs (±4 mm) are reported. Other conventions as in Figs 2 and 3.

Hand/finger motor skills were tested in all participants on the same day of fMRI acquisition, using the Purdue Pegboard Test (PPT)^26^ (see Methods). The PPT scores were entered in an fMRI regression analysis to test for a possible relationship between the degree of individual hand motor dexterity and brain activity associated with the observation of complex hand-object manipulative actions performed by the skilled model. BOLD signal change in three of four identified ROIs belonging to the AON (Fig. 6A) positively correlated with the PPT scores obtained by participants using the right dominant hand (Fig. 6B). During the observation of the expert model performing complex manipulative actions, subjects demonstrated a significant positive correlation between BOLD signal change in the left SPL (*r* = 0.52, *P* < 0.01), left IPS (*r* = 0.47, *P* < 0.05) and left PMd (*r* = 0.56, *P* < 0.01). On the contrary, there were no significant correlations between PPT individual scores and BOLD activity in PMv (*r* = 0.07, *P* > 1, n. s.).

### PPI analysis

**Figure 7:**
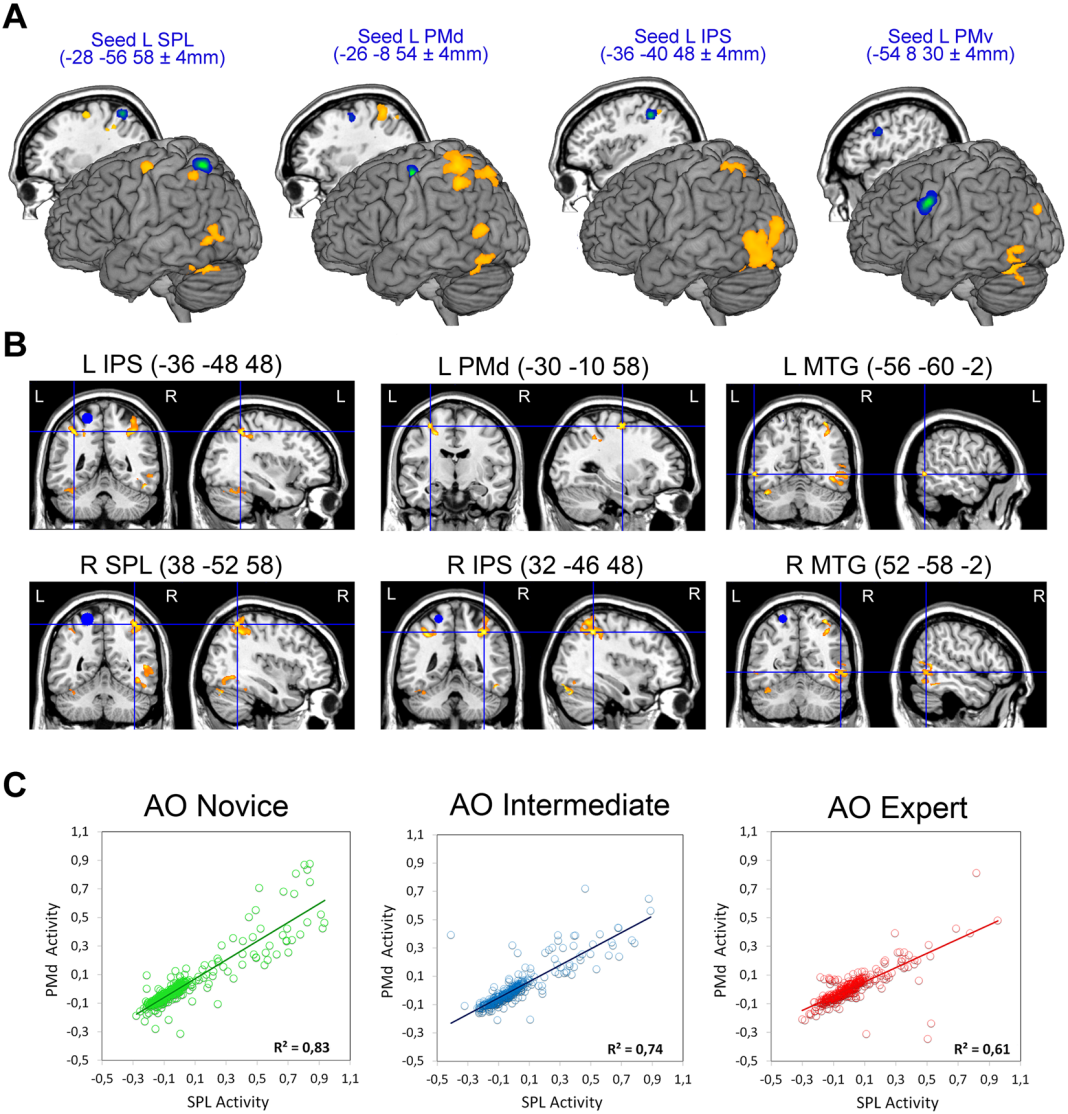
PPI analysis results. (**A**) Overlap maps of SPL, PMd, IPS and PMv individual seed regions used for PPI analysis, rendered on a standard 3D MNI brain template (green-blue color scale) and the corresponding areas functionally connected with them (orange color). Behind to each render, parasagittal sections show medial location of seeds overlap. Mean MNI coordinates for individual seeds taken for PPI are reported on the top of each render. (**B**) Brain regions increasing their functional connectivity with SPL seed region during the observation of novice model compared to the observation of expert. Activation clusters are shown at *P* < 0.001, FWE corrected at cluster level. (**C**) Correlations between brain activity (BOLD signal change) in SPL region and PMd during the three action observation experimental conditions.

To determine which brain areas have a specific role in motor resonance induced by the observation of actions performed with high level of manual expertise, we adopted a functional connectivity approach by using a psycho-physiological interaction (PPI) analysis^27,28^ (see Methods), requiring a PPI between a seed region and the whole-brain, voxel-by-voxel. In particular, we conducted four different PPIs to test the hypothesis that functional connectivity between areas in the parieto-premotor AON would be greater when a participant observe actions already embodied in the personal own motor repertoire, like daily life actions that the individual can perform, as compared to complex skilled actions. As seed regions, we defined at single subject level four volumes of interest (VOIs) (4-mm radius sphere), using a conjunction analysis between the main action observation conditions, masked with an inclusive contrast image corresponding to *Act_Exe*, around local maxima in the left SPL (x = −28, y = −56, z = 58; SD ± 4 mm), left PMd (x = −26, y = −8, z = 54; SD ± 4 mm), left IPS (x = −36, y = −40, z = 48; SD ± 4 mm) and left PMv (x = −54, y = 8, z = 30; SD ± 4 mm). This yielded 18/18 participants with valid seed regions. As shown in Fig. 7A–C, left SPL demonstrated significantly increased functional coupling with clusters in the left hemisphere as left PMd cortex (peak: *x* = −30, *y* = −10, *z* = 58; *t* = 5.88; *P = *0.03, False Discovery Rate (FDR) corrected), left IPS (peak: *x* = −36, *y* = −48, *z* = 48; *t* = 6.12; *P = *0.006, FDR corrected), left MTG (peak: *x* = −56, *y* = −60, *z* = −2; *t* = 6.05; *P = *0.02, FDR corrected) (see Suppl. Table S2). Moreover, left SPL was functionally connected with areas in the right hemisphere as right SPL (peak: *x* = 38, *y* = −52, *z* = 58; *t* = 5.60; *P = *0.0001, FDR corrected), right IPS (peak: *x* = 32, *y* = −46, *z* = 48; *t* = 6.48; *P = *0.001, FDR corrected) and right MTG (peak: *x* = 52, *y* = −58, *z* = −2; *t* = 4.92; *P = *0.001, FDR corrected). Interestingly, the PPI analysis performed using as seed the PMd region revealed increased connectivity within dorsal but not ventral areas of the AON, including left SPL (peak: *x* = −28, *y* = −54, *z* = 60; *t* = 5.40; *P = *0.005, FDR corrected), left IPS (Area hIP2; peak: *x* = −46, *y* = −46, *z* = 46; *t* = 4.55; *P = *0.01, FDR corrected) and right IPS (Area hIP3; peak: *x* = −36, *y* = −50, *z* = 50; *t* = 4.45; *P = *0.005, FDR corrected) (see Fig. 7A).

The third PPI analysis carried out using as seed IPS did not show any effective connectivity with ventral AON areas during AO Novice vs Expert, but only with SPL bilaterally (left SPL peak: *x* = −46, *y* = −52, *z* = 52; *t* = 4.86; *P = *0.005, FDR corrected; right SPL (peak: *x* = 22, *y* = −68, *z* = 60; *t* = 4.78; *P = *0.02, FDR corrected). Finally, using PMv as seed for PPI we did not find any correlation with voxels in the parieto-premotor AON. Only voxels in the MTG (peak: *x* = −48, *y* = −58, *z* = 10; *t* = 5.38; *P = *0.01, FDR corrected) survived.

This demonstrates that the observation of actions performed by the novice model, already present in the motor repertoire of participants, not only activates the AON but also modulates the connectivity between left SPL and the other dorsal parieto-premotor areas. On the contrary, no functional modulation was present in the inferior frontal areas considered part of the ventral parieto-frontal MNS/AON, like PMv.

## Discussion

The aim of this study was to determine, in a group of healthy individuals without specific motor skills, whether the observation of actions performed with different levels of expertise modulates the activations of areas belonging to the AON. Thus, the employed paradigm, in contrast to previous neuroimaging studies, was not limited to comparing brain activations of a specialized population of expert versus non-expert individuals, but to assessed whether in naïve subjects motor ‘resonance’ is modulated by higher or lower similarity between the observer’s motor repertoire and that of the observed model. Our results showed that the SPL and PMd regions of participants were activated more strongly during observation of complex object manipulation performed by a naïve model without particular hand skills than during observation of more skilled models (intermediate and expert). Moreover, the single-subject BOLD activation within the same regions during observation of actions performed by the expert was positively correlated with her/his hand dexterity, as assessed through a standardized behavioral motor test. Finally, the psychophysiological interaction analysis during observation of the naïve model compared with that of the expert, revealed enhanced functional connectivity between dorsal areas of the AON, including the SPL and other regions anatomically and functionally coupled to it, such as the PMd cortex, compared to the ventral parietal and premotor areas. Altogether, these data suggest a possible distinction between ventral and dorsal brain circuits in the processing of different aspects of action perception.

The modulation of AON activation during observation of complex manipulative actions performed with different levels of expertise could be attributed to the basic visual features of the employed stimuli or to spurious factors. The ROI analysis carried out in visual areas V1 and MT/V5 in order to control for the impact of early visual effects did not show any significant difference between conditions. Another possible confound could be the actual duration of the observed manipulation, which was dissimilar across the three conditions. However, the absence of a difference in motion energy quantification between conditions seems to exclude this explanation. Furthermore, the introduction of stimulus duration as factor in the first-level analysis showed an effect only in V4, and no effect was present in parietal and premotor areas, confirming that temporal exposure to these stimuli was not able to explain the main result. Another possible confounding factor could be the visual familiarity with the observed model. However, all observed models were unfamiliar to the participants, because they had not previously seen any of the presented actions. Our findings could be also due to a different degree of attractiveness of the expert model, because in a post-acquisition debriefing interview, the participants confirmed that the expert actor was ‘attractive’. However, if the model’s attractiveness had generated higher levels of attention or general arousal, we should have observed higher activation during AO Expert. We found exactly the opposite. Thus, the crucial factor that can explain our findings appears to be the different levels of expertise exhibited by each model; this conclusion is in line with the literature on the relationship between motor experience and activity in the cortical motor system during action observation^19–21,23,24^. Notably, in the present study, because the participants were naïve with respect to the observed action performed with high dexterity, the strongest activation occurred during observation of the low-level performance that better corresponded to their motor repertoire. In addition, because our paradigm also included an intermediate level of ability, we were able to demonstrate that the motor resonance induced by action observation is not an all-or-nothing process but involves different levels of mirroring of the observed model.

By comparing the activations induced by the observation of manipulative actions performed by the novice versus the expert, we found activation in a dorsal network of areas constituted primarily by the SPL and PMd, selectively modulated by the actor’s motor expertise. This finding emerged also from the PPI analysis performed to highlight, in the whole-brain, voxels functionally coupled with the SPL and PMd seed regions, during the observation of naïve actions. These results are partially different from those of previous neuroimaging and neurophysiological studies in humans, which have suggested that the AON primarily encodes the goal of the observed actions. For instance, studies on the observation of actions performed by humans versus artificial agents (e.g. a robotic arm), have shown a similar activation of the AON, although these types of effector differ in terms of both appearance and kinematic features^29,30^.

On the other hand, our findings are in line with studies demonstrating that the human AON is activated not only by goal-directed actions but also by intransitive finger or arm movements, thus indicating a sensitivity to kinematic parameters^5,31–33^. For instance, by comparing BOLD responses during the observation of intransitive movements complying with or violating the two-thirds power law of the movement, Casile *et al*.^33^ showed higher activations, for the former, within the PMd, middle superior frontal gyri and medial frontal cortex. Starting from the assumption that several mechanisms can be involved in action observation, Hamilton and Grafton carried out two studies^34,35^. In the first, they showed that the aIPS is more involved in goal processing, whereas the left lateral occipital sulcus and right PMd are more involved in encoding movement trajectory. In the second, they showed that the *action outcomes* activate more the bilateral IPL and IFG (ventral AON), whereas the *means* are encoded in the left SPL, left middle IPS, left occipito-temporal cortex and STS. These findings support the idea that dorsal regions of the AON can be involved in encoding the way in which movements are executed. In this study, we used only one type of action, characterised by a sequential manipulation of an object from the thumb to the little finger, while the main variable consisted in the actors’ hand motor dexterity. Thus, it is very likely that the differences observed in the activation among the conditions result from differences in action kinematics patterns. The results suggest that the dorsal AON, in contrast to the ventral AON, may be modulated by movement kinematics, because its activation was different depending on the observed action skill. Interestingly, this difference was also confirmed by PPI analysis, showing that the PMv, differently from the PMd, does not show any functional connectivity with SPL, suggesting that the dorsal and ventral premotor cortices play different roles in motor resonance induced by observed action.

Previous studies have distinguished the contribution of dorsal and ventral parieto-frontal networks as separate modules for reaching and grasping [see^36–38^]. In fact, in the monkey, reaching movements are processed within the so-called dorso-dorsal circuit^39,40^, whereas grasping movements are processed within the dorso-ventral circuit^41–43^. Similarly, in humans the execution of reaching movements is typically associated with the activation of the dorsal circuit (SPL and PMd)^17,18,44,45^, whereas grasping mostly activates the ventral circuit (anterior IPS and PMv/IFG)^25,46,47^. Note that the same pattern of dorsal activation was found also during pure observation of goal-directed reaching movements^17,18,47^.

However, over the last decade, a series of studies have shown that the monkey parietal area V6A and dorsal premotor area F2 are involved in visually guided reaching-to-grasp movements^48–52^. Thus, this evidence may provide an explanation for our data, suggesting that not only the ventral but also the dorsal circuit is involved in hand motor control. More specifically, the activation of the dorsal circuit may reflect the encoding of some kinematic aspects of manipulative actions (including direction of movement, speed and acceleration), particularly when online control and corrections are needed^52,53^. In the context of study, it is plausible that the observation of an actor with similar hand ability activated regions in the dorsal network, as though participants needed to learn the observed complex actions by decoding their kinematic features as well.

Accordingly, several single neuron studies in monkeys have demonstrated that kinematic parameters are encoded in both the dorsal premotor^54–58^ and superior parietal^59–62^ cortex. Note however, that all the studies described above investigated arm movements; to our knowledge, there are no reports of activation of single neurons in the dorsal circuit associated to hand kinematics processing.

In conclusion, we hypothesise that the dorsal AON is likely involved in the kinematic analysis of observed hand motion, thus playing a relevant role in hand movement planning and selection^63^. These properties could have important applications in observational learning of new hand motor abilities and in observation-based rehabilitation for the recovery of manual ability, where imitation of an observed model requires a preliminary coding of both action goal and kinematic properties^64^.

## Methods

### Participants

Eighteen human volunteers (10 females; mean age 22.5 years; range18–25 years) with normal or corrected-to-normal vision, no history of neurological, orthopedic or rheumatologic disorders, and no drug or alcohol abuse participated in the study. All participants were right-handed according to the Edinburgh Handedness Inventory^65^. Subjects with particular manual ability (e.g., musicians, athletes, typewriting, etc.) were not included in the study. All participants were selected on the basis of their daily activities, in terms of hobbies and amusements. The study was approved by the local ethics committee (Comitato Etico per Parma, University of Parma) and participants gave their informed written consent in accordance with the Declaration of Helsinki.

### Hand motor skills Assessment

The Purdue Pegboard Test (PPT)^26^ consists of a board with two parallel rows made of 25 holes per row into which cylindrical metal pins have to be placed by the participant. After explanation, as well as demonstration of the task and three practice trials, participants were asked to place as many pins into the holes of the perforated board as possible within 30-s of each trial. Three trials for each conditions were administered, that is with the dominant (right) hand, with the non-dominant hand and with both hands; manual ability scores (PPT scores) were obtained by averaging the number of pins correctly placed during each condition (we consider for the successive analysis only the right-dominant hand PPT score). Individual normalized PPT scores are reported in Supplementary Table S3.

### Scanning procedure, Stimuli and Tasks

Participants went through a training phase before fMRI scanning aimed at familiarizing them with the experimental procedure. They laid supine in the bore of the scanner in a dimly lit environment. Visual stimuli were presented in the fronto-parallel plane by means of a digital video system (60 Hz refresh rate) with a resolution of 800 horizontal pixels × 600 vertical pixels with horizontal eye field of 30° (Resonance Technology, Northridge, CA). Sound-attenuating (30 dB) headphones were used to muffle scanner noise and give instructions to subjects. Digital transmission of signal to scanner was via optic fiber. Software E-Prime 2 Professional (Psychology Software Tools, Inc., http://www.pstnet.com) was used both for stimulus presentation and recording of participant response. Before the beginning of the MRI acquisition, subjects received the instruction as not to make any voluntary movement and only concentrate on the video screen. Absence of actual hand movement during tasks was visually checked by the investigator.

Movies were recorded using a digital HD Camera and edited using AdobePremiere software (www.adobe.com). Supplementary Videos 1–6 represent examples of action presented to participants during fMRI, showing object manipulation performed by the naïve model (Supp. Videos 1 and 2), by the model with intermediate level of hand motor skills (Supp. Videos 3 and 4) and by the expert model (Supp. Videos 5 and 6). General actors hand motor skill was assessed using the PPT test, the Maximum finger tapping frequency and the Minnesota Dexterity Test^66^. Individual mean and equivalent scores related to actor’s performances are reported in Supplementary Table S4. The actions were presented in a first-person perspective, according with the literature on the monkey MNS, reporting that during observation of actions performed from different viewpoints there is a tendency for a higher number of mirror neurons preferring the subjective perspective with respect to third person perspective^67^. The objects used for manipulation were a ball, a cylinder or a coin. Each action was recorded twice to take into account the variability of the execution performance. A total of 18 experimental video stimuli (3 actors × 3 manipulated objects × 2 repetitions) and 18 control video stimuli were presented in the action observation task. For motion energy quantification, see Supplementary Information. Experimental and control conditions were presented in independent 15 s blocks (see Fig. 1B), constituted by five randomly presented videos (3 s each). Each run was arranged to include a total number of 16 blocks, 4 blocks for each condition. Blocks of stimuli were interleaved by a fixation–no clip event (*rest*) lasting 9–15 s, used as baseline. In 25% of the blocks, participants had to provide an explicit response (catch-trials), using a response pad placed inside the scanner, concerning the shape of the object observed in the last video clip, thus unrelated to motor aspects of the task. The catch-trials were randomly presented and lasted 3 s each, followed by a 12 s *rest* period to remove artefacts derived from the motor component. Before performing the Localizer, subjects were familiarized with the table (40 × 30 cm) to be placed on her/his lap during scanning. The table contained a square box with a small wooden cylinder inside. The task was performed without visual feedback of the own hand, since the subjects were presented with visual instruction cues, by means of digital goggles system, similarly to the action observation task. Each action started from the same position and terminated in the same final position. The task sequence was as follows. At the beginning of each block, an instruction cue (orange fixation cross) was presented for 2 s on the black screen, in order to control for movement preparation (action preparation, *Act Pre*). After 2 s, fixation cross color turned to green instructing subject to perform object manipulation (action execution, *Act Exe*). The baseline condition (*rest*) consisted of the static presentation of a white cross in the middle of the screen. The task was performed alternating experimental blocks and baseline period in a counterbalanced manner. Each of the action observation runs lasted about 8 min. The Localizer run lasted about 5 min.

Imaging parameters

See Supplementary Information.

fMRI Data preprocessing and analysis

See Supplementary Information.

### ROI-based analysis

Regions of interest (Fig. 5A) were defined in subject’s space before normalization, according to the following criteria: (1) activated voxels overlapped for the four experimental and control conditions (*AO Novice*, *AO Intermediate*, *AO Expert and AO Ctrl*) masked with the contrast image derived from the action execution Localizer (*Act Exe* vs *rest*); (2) the individual masked overlapping activations had to survive a threshold of *P* < 0.001, FWE corrected at cluster level; (3) voxels were in anatomical regions identified in the literature as being involved in action observation^5,18^ namely, superior parietal lobule (SPL), intraparietal sulcus (IPS), dorsal premotor cortex (PMd) and ventral premotor cortex (PMv). For each subject we defined a sphere, with maximum cluster size of 4 mm radius around the peak activation, within each anatomically defined region. After, we mapped individual ROIs on a standard MNI template in order to obtain standard coordinates (see Suppl. Table S5) and overlap. Regions revealed in this analysis include left SPL (ROI 1: x = −28, y = −56, z = 58; ± 4 mm), left PMd (ROI 2: x = −26, y = −8, z = 54; ± 4 mm), left IPS (ROI 3: x = −36 y = −40 z = 48; ± 4 mm) and left PMv (ROI 4: x = −54, y = 8, z = 30; ± 4 mm respectively. An additional control ROI analysis was carried out in visual areas V1 and MT/V5, bilaterally, in order to exclude possible variations in BOLD signal change for different experimental conditions in early or associative visual areas. Anatomical descriptions were, in the majority of the cases, based on the probabilistic cytoarchitectonic maps of the brain mapping group in Juelich (Germany), as implemented in the SPM anatomy toolbox (http://www.fz-juelich.de/ime/spm_anatomy_toolbox)^68^. Then, for each subject we calculated separately in each ROI the average BOLD signal change across all significant voxels using the SPM Rex Toolbox (http://web.mit.edu/swg/rex).

### Regression Analysis

To investigate the relationship between functional activations changes within the MNS areas and the level of own finger/manual dexterity assessed with the PPT we performed first a conjunction analysis to highlight voxels activated either during the action observation of expert model (*AO Expert* vs *rest*) and action execution (*Act Exe* vs *rest*). Using the conjunction analysis map, four ROIs were defined centring the sphere (radius 4-mm) at the local maxima. Then, the BOLD signal change associated with the observation of expert model was extracted in the four ROIs for each subject. It is important to note that the individual behavioural score entered in the regression analysis was related exclusively to the execution of the PPT task performed using the right dominant hand, spatially compatible with that observed by participants from a first-person perspective during the fMRI action observation task.

### Psychophysiological Interaction (PPI) Analysis

We performed a psychophysiological interaction analysis (PPI)^27,28^ to estimate the functional connectivity between brain areas involved in the processing of complex hand-object manipulation during the observation of a novice model versus an expert one. The purpose of PPI analysis is to determine which voxels in the whole-brain increase their relationship with a seed region of interest in a given context, such as during a particular behavioral task. In other words, PPI aims to identify regions whose activity depends on an interaction between psychological and physiological factors (the BOLD time course of a region of interest). A task-specific increase in the relationship between brain regions (a PPI effect) is suggestive of an increase in the exchange of information^28^. In this study, we performed four PPI analyses in order to highlight brain regions whose activity depends on an interaction between observation of novice versus expert model (Psychological task) and the BOLD signal change in four ROIs defined individually in left SPL, PMd, IPS and PMv (Physiological response). For each ROI, the PPI procedure was performed at the single-subject level in 3 different steps: (1) extraction of the BOLD signal in a seed ROI; (2) PPI analysis; (3) PPI GLM analysis.

#### Step 1

For each participant, the seed region (4 mm radius sphere) was localized around local maxima defined using the previous ROI analysis in the left SPL (x = −28, y = −56, z = 58; SD ± 4 mm), left PMd (x = −26, y = −8, z = 54; SD ± 4 mm), left IPS (x = −36, y = −40, z = 48; SD ± 4 mm) and left PMv (x = −54, y = 8, z = 30; SD ± 4 mm). This yielded 18/18 participants with valid seed regions. Then, using the SPM12 eigenvariate function, we extracted for each subject the time course of activity in the volume of interest (VOI).

#### Step 2

The PPI analysis employed 3 regressors as follows: one regressor represented the deconvolved activation time course^27^ in the seed (Y vector, physiological variable), one regressor represented the contrast of interest [AO Novice (t) vs AO Expert (t)] (P vector, psychological variable), and one regressor represented the interaction of the previous two vectors (PPI, the interaction term). PPI analysis assessed the connectivity between seed and whole-brain by multiplying the deconvolved BOLD signal with the psychological vector.

#### Step 3

After deconvolution of HRF, for each participant, the 3 regressors (PPI term, Y vector, and P vector) were entered into a first-level GLM to determine which brain regions increased their connectivity with each seed. As covariates of no interest, models also included the six motion parameters. To investigate PPI effects at multi-subject level, we entered contrast images of the PPI effects for each participant into a random-effects analysis using a flexible factorial repeated-measures ANOVA (within-subject factor: level of expertise; blocking factor: subject).

## Electronic supplementary material

Supplementary Video 1

Supplementary Video 2

Supplementary Video 3

Supplementary Video 4

Supplementary Video 5

Supplementary Video 6

Supplementary Information
