# Supplementary material for: Parieto-frontal mechanisms underlying observation of complex hand-object manipulation
Source: Sci Rep. 2019 Jan 23;9:348. doi: 10.1038/s41598-018-36640-5 (PMC6344645; doi:10.1038/s41598-018-36640-5)
Supplement: Supplementary file 7 — Supplementary Information [file 41598_2018_36640_MOESM7_ESM.pdf]

Supplementary Information

Title

Parieto-frontal mechanisms underlying observation of complex hand-object manipulation

Authors

Antonino Errante[1] and Leonardo Fogassi[1*]

[1] Department of Medicine and Surgery, University of Parma, Parma, 43125, Italy

Motion Energy Quantification

Since video clips related to the main experimental conditions *AO Novice*, *AO Intermediate* and *AO Expert* varied in the amount of finger motion and velocity profiles (intrinsic features of the three categories of expertise) it was necessary to control that differences observed in brain activation were not due to low-level visual features. To take into account the amount of visual information between categories, we quantified the motion energy in each video clip using Matlab algorithm VIP Motion working on Simulink (Mathworks, Inc., Natick, MA). Such quantification of motion energy employs motion detecting method [S1]. Similar methods were previously used in other neuroimaging studies on action observation [S2, S3]. We calculated the sum of absolute differences (SAD) between two consecutive frames of each video clip within a category, to measure the similarity between blocks of frames-images. SAD was calculated by taking the absolute difference in the red, green, and blue channels between each pixel in the original block and the

corresponding pixel in the block being used for comparison. These differences were summed to create a simple metric of block similarity. The ANOVA results showed no significant difference ($F_{2, 5} = 0.159$, $P < 0.854$) between pixels changing per frame computed for actions performed by novice (M = 3002), intermediate (M = 2905) and expert (M = 2893).

Imaging parameters

Anatomical T1-weighted and functional T2*-weighted MR images were acquired with a 3 T General Electric scanner equipped with an 8-channel receiver head-coil. A three-dimensional (3D) high-resolution T1-weighted IR-prepared fast SPGR (Bravo) image covering the entire brain was acquired in one of the scanning sessions and used for anatomical reference. Its acquisition parameters were as follows: 196 slices, 280×280 matrix with a spatial resolution of 1×1×1 mm, TR = 9700 ms, TE = 4 ms, FOV = 252 x 252 mm; flip angle = 9°. Functional volumes were acquired while participants performed the action observation task and the action execution Localizer with the following parameters: thirty-seven axial slices of functional images covering the whole brain acquired using a gradient-echo echo-planar imaging (EPI) pulse sequence, slice thickness = 3 plus interslice gap = 0.5 mm, 64×64×37 matrix with a spatial resolution of 3.5×3.5×3.5 mm, TR = 3000 ms, TE = 30 ms, FOV = 205 x 205 mm^2, flip angle = 90°, in plane resolution = 3.2 x 3.2 mm^2.

fMRI data Preprocessing

Data processing was performed with SPM12 (Wellcome Department of Imaging Neuroscience, University College, London, UK; http://www.fil.ion.ucl.ac.uk/spm) running on MATLAB R2016 (The Mathworks, Inc.). Structural images were manually centered and reoriented with functional images to the anterior-posterior commissure axis. The first four EPI volumes of each functional run

were discarded to allow the magnetization to reach a steady state. For each subject, all volumes were slice timing corrected, spatially realigned to the first volume of the first functional run and un-warped to correct for between-scan motion. Motion parameters were used as predictors of no interest in the model to account for translation and rotation along the three possible dimensions as determined during the realignment procedure. Individual dataset was excluded if excessive head motion was observed (translation > 3 mm or rotation > 3°). T_1-weighted image was segmented into gray, white and cerebrospinal fluid and spatially normalized to the Montreal Neurological Institute (MNI) space. Spatial transformation derived from this segmentation was then applied to the realigned EPIs for normalization and re-sampled in $2 \times 2 \times 2$ mm^3 voxels using trilinear interpolation in space. All functional volumes were then spatially smoothed with a 6-mm full-width half-maximum isotropic Gaussian kernel.

fMRI Statistical analysis

Data were analysed using a random-effects model [S4] implemented in a two-level procedure. In the first-level, single subject fMRI responses were modelled using a General Linear Model (GLM), for which a design-matrix included the onsets and durations of each event for each condition. The model combined the two action observation runs, modelling five predictors corresponding to experimental action observation conditions, control condition and response to catch-trials (*AO Novice*, *AO Intermediate*, *AO Expert*, *AO Ctrl and Response*), six predictors obtained from the motion correction in the realignment process to account for voxel intensity variations caused by head-movement and one constant regressor per run. We included also actual *Stimulus duration* (ms) as a regressor in the GLM, in order to investigate the impact of temporal exposition to different experimental conditions. All predictors, except for *Response* and *Stimulus duration*, included the five consecutive videos of each trial, which were modelled as one single epoch lasting 15s. Catch trials were modelled as consecutive blocks, lasting 15s each, including the effective response time (3s) and a signal-denoising period (12s) to separate the motor component from subsequent

processing. In the second level group-analysis, corresponding *t*-contrast images (*AO Novice* vs *rest*, *AO Intermediate* vs *rest*, *AO Expert* vs *rest*, *AO Ctrl* vs *rest*) of the first-level models were entered in a flexible ANOVA with sphericity-correction for repeated measures [S5]. Within this model, we also assessed the activations resulting from the direct contrasts between conditions (*AO Novice* vs *AO Ctrl*, *AO Intermediate* vs *AO Ctrl*, *AO Expert* vs *AO Ctrl*, and all reverse contrasts) with a statistical threshold of $P < 0.001$, with family wise error (FWE) correction at cluster level. Finally, in order to assess brain region modulated by expertise effects, we computed direct contrasts between conditions (*AO Novice* vs *AO Intermediate, AO Novice* vs *AO Expert* and all reverse contrasts) with a statistical threshold of $P < 0.001$ family wise error (FWE) corrected at cluster level). Data corresponding to the action execution Localizer were analysed using a GLM with two predictors (*Act Pre* vs *rest*, *Act Exe* vs *rest*) convolved with the HRF. This latter contrast was used for the localization of regions of interest (ROIs, see next section) belonging to the parieto-premotor MNS, involved also in both action execution and observation. Results of the Localizer were thresholded at $P < 0.05$, FWE corrected for multiple comparisons at the voxel level for GLM univariate analysis, or $P < 0.001$ at the cluster level for the localization of individual activation foci within the parieto-premotor MNS. Local maxima of activations are presented in the stereotaxic space of the MNI coordinate system.

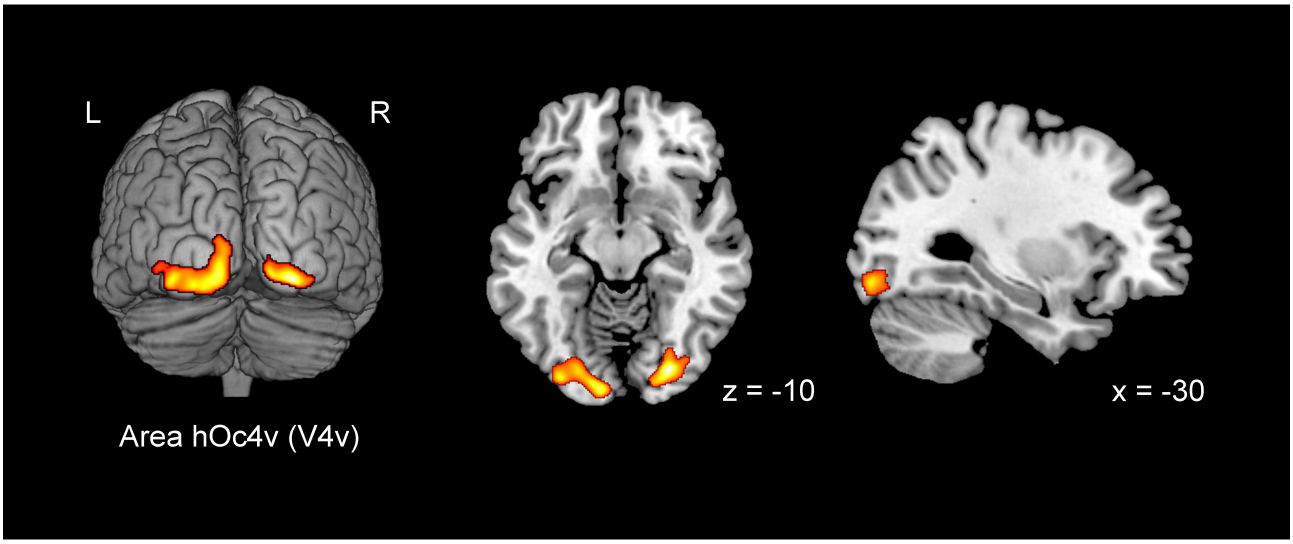

Supplementary Figure S1. Statistical map showing areas whose activity is explained by actual duration of video-stimuli in different experimental conditions (AO Novice, AO Intermediate, AO Expert). The activations are rendered into a standard MNI brain template (*P* < 0.001, FWE corrected at cluster-level).

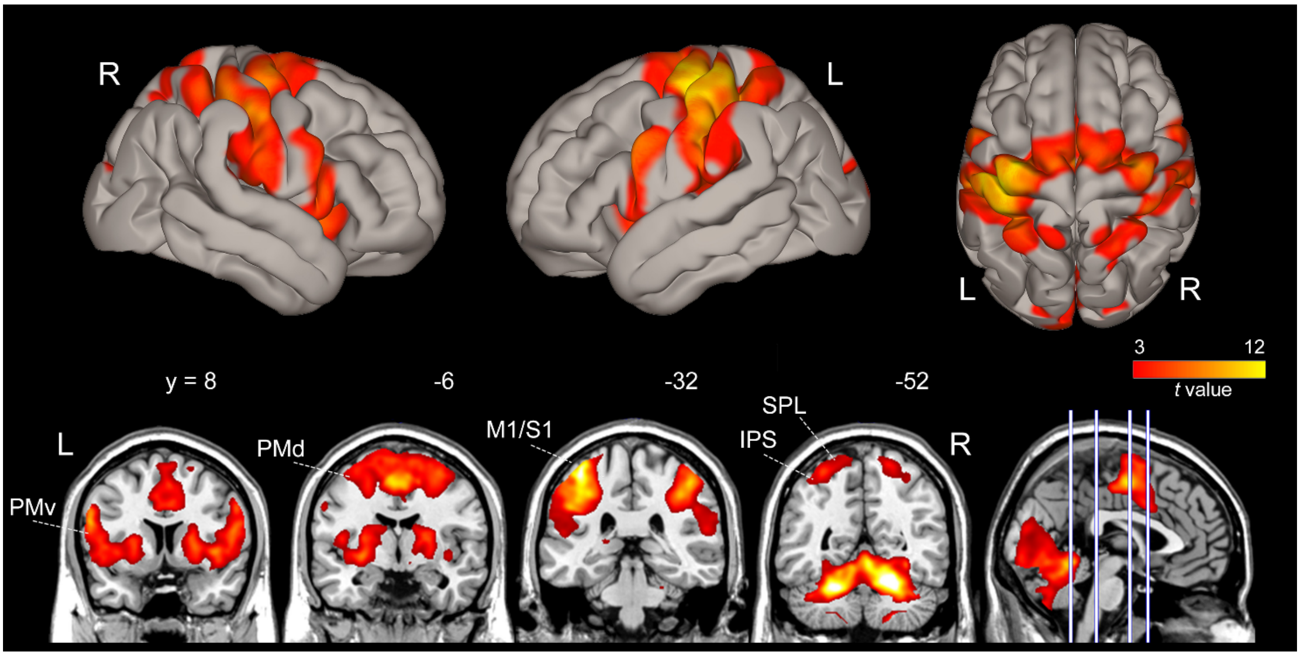

Supplementary Figure S2. Brain activation map resulting from action execution Localizer (*Act Exe* vs *rest*). The activations are rendered into a standard MNI brain template ($P < 0.05$, FWE corrected at voxel-level). Vertical lines on the parasagittal view indicate approximate locations of the four displayed different coronal sections. Abbreviations: IPS, intraparietal sulcus; M1, primary motor cortex; PMd, dorsal premotor cortex; PMv, ventral premotor cortex; S1, primary somatosensory cortex; SPL, superior parietal lobule. Other conventions as in Fig. 2.

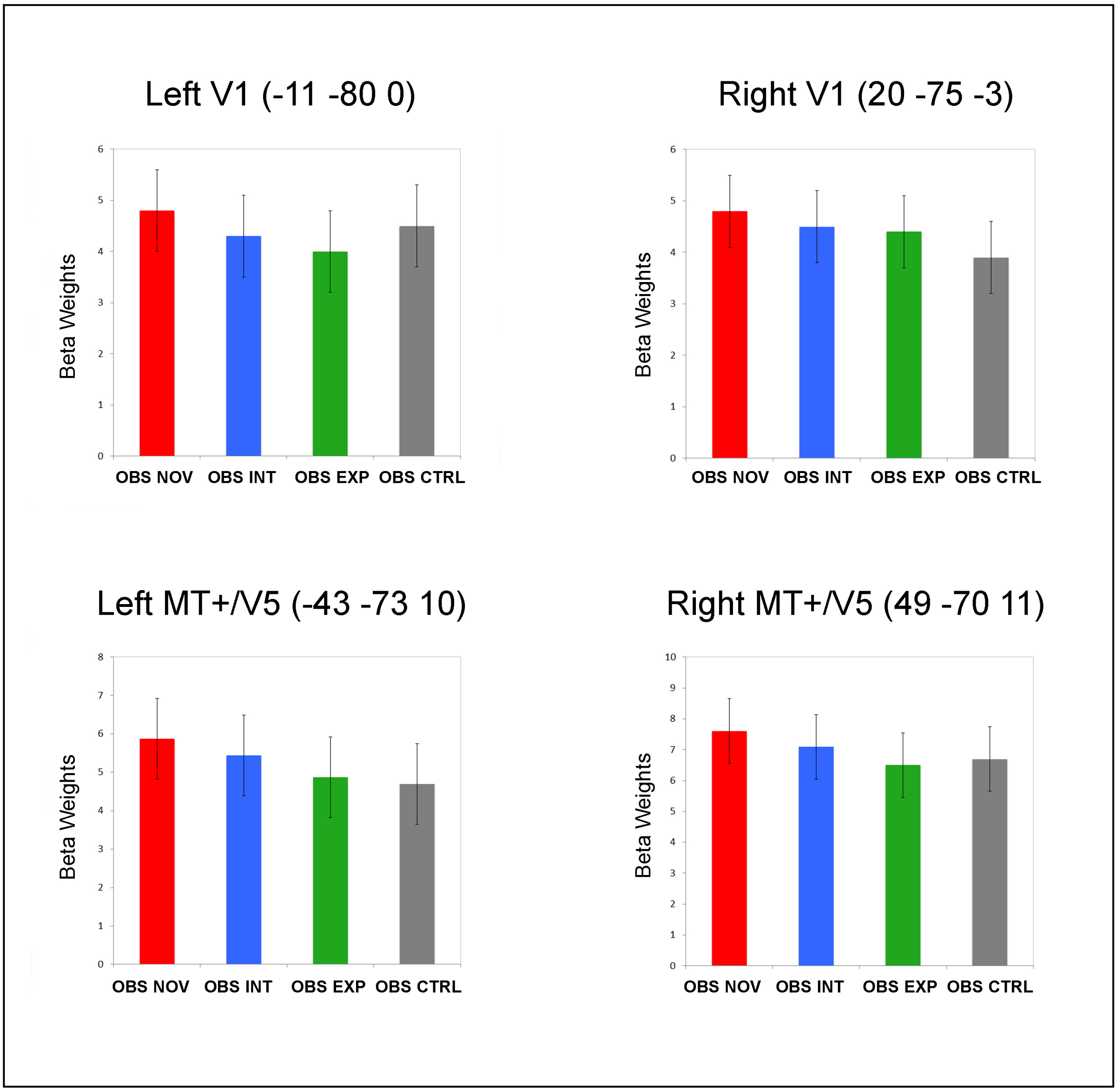

Supplementary Figure S3. Results of the control ROI analysis. Histograms showing the averaged magnitude of activation (parameter estimate) in each visual ROI for both experimental and control conditions.

Anatomical region	Left Hemisphere				Right Hemisphere			
	x	y	z	Z-score	x	y	z	Z-score
**AO Novice vs AO Ctrl**								
Inferior Parietal Lobule (PFt)	-50	-22	32	7.10	62	-22	30	6.12
Cerebellum (LobuleVI)	-28	-54	-18	5.96	28	-54	-20	6.73
Precentral gyrus	-30	-12	64	6.51	28	-10	58	6.14
Insula	-38	-6	14		38	0	11	6.33
Cingulate Cortex	-6	-30	24	5.15	6	-22	26	6.06
Intraparietal Sulcus	-34	-54	56	3.70	40	-44	50	4.38
Thalamus					26	-26	0	4.88
IFG (pars Triangularis)					40	32	16	4.63
IFG (pars Opercularis)					58	14	32	4.51
Fusiform gyrus	-30	-52	-14	6.25	34	-48	-14	6.47
Superior Parietal Lobule	-32	-46	64	6.16	34	-52	60	5.51
Postcentral Gyrus	-38	-38	56	6.07	32	-36	52	6.14
**AO Intermediate vs AO Ctrl**								
Inferior Parietal Lobule (PFt)	-50	-22	32	6.53	53	-20	30	5.32
Cerebellum (LobuleVI)	-28	-50	25	6.51				
Precentral gyrus	-30	-14	62	6.09	30	-10	58	5.10
Cingulate Cortex	4	-28	30	6.17				
Middle Frontal Gyrus					48	40	22	3.82
IFG (pars Triangularis)					48	22	24	3.40
Thalamus					12	-22	-8	3.42
Fusiform gyrus	-30	-50	-14	6.46	30	-48	-14	7.08
Superior Parietal Lobule					34	-52	60	4.44
Postcentral Gyrus	-38	-38	58	5.20	32	-36	52	5.37
Precuneus	-14	-64	34	5.08	14	-70	40	5.88
**AO Expert vs AO Ctrl**								
Inferior Parietal Lobule (PFt)	-64	-26	34	4.67	54	-22	30	4.56
Cerebellum (LobuleVI)	-28	-50	-24	4.81	26	-54	-20	4.96
Precentral gyrus	-30	-12	64	4.40				
Insula					38	0	12	3.96
Cingulate Cortex	-4	-20	30	4.64	6	-14	30	4.76
IFG (pars Triangularis)					40	34	12	4.43
Fusiform gyrus	-30	-52	-14	4.62	26	-62	-10	5.33
Superior Parietal Lobule					34	-52	60	3.57
Postcentral Gyrus	-42	-30	42	4.21	50	-24	46	3.55
Precuneus	-14	-65	34	3.54	12	-66	36	4.65

Supplementary Table S1. Statistical values for GLM group analysis related to the action observation task. Local maxima corresponding to the activation maps shown in Figure 3 are given in MNI standard brain coordinates. Significant threshold is set at $P < 0.001$, FWE-corrected at cluster-level.

Anatomical region	Left Hemisphere				Right Hemisphere			
	x	y	z	Z-score	x	y	z	Z-score
**A) PPI Left SPL**								
Superior Parietal Lobule	-	-	-	-	38	-52	58	3.99
Intraparietal Sulcus	-36	-48	48	4.20	32	-46	48	4.34
Precentral Gyrus	-30	-10	58	4.11				
Postcentral Gyrus	-34	-34	42	3.72				
Cerebellum (Crus 1)	-40	-60	-26	4.18	34	-82	-20	4.57
Middle Temporal Gyrus	-56	-60	-2	4.17	52	-58	-2	4.21
**B) PPI Left PMd**								
Superior Parietal Lobule	-28	-54	60	5.40				
Intraparietal Sulcus (Area hIP2)	-46	-46	46	5.36				
Intraparietal Sulcus (Area hIP3)					36	-50	50	3.57
Middle Temporal Gyrus (MT/V5)	-46	-66	10	3.49				
**C) PPI Left IPS (Area hIP3)**								
Superior Parietal Lobule	-28	-54	60	5.39	22	-56	62	5.43
Middle Temporal Gyrus (MT/V5)	-46	-72	-2	5.34	52	-62	-6	5.12
**D) PPI Left PMv**								
Angular Gyrus	-36	-50	-24	4.24				
Inferior Temporal Gyrus	-50	-50	-22	3.43				

Supplementary Table S2. PPI activations. Brain areas involved in a psychophysiological interaction with SPL, PMd, IPS and PMv seeds during the observation condition AO Novice vs AO Expert, with a threshold set at $P < 0.001$, FWE-corrected at cluster level. Local maxima, as in Figure 7, are given in MNI standard brain coordinates.

Sub Nr.	*Left Hand (Z)*	*Right Hand (Z)*	*Bimanual (Z)*
# 1	-0.80	-0.81	0.48
# 2	-0.03	-0.89	0.48
# 3	-0.61	-0.21	2.51
# 4	0.03	0.41	1.33
# 5	0.55	1.33	1.60
# 6	-0.15	0.19	1.50
# 7	0.67	0.43	1.65
# 8	1.26	-2.24	0.21
# 9	-0.61	-0.71	1.24
# 10	-0.18	0.08	0.55
# 11	-0.16	0.21	1.13
# 12	-1.02	-1.45	-1.78
# 13	-1.67	-2.29	0.48
# 14	-0.37	-0.57	-0.87
# 15	-0.58	0.21	0.81
# 16	0.39	-0.04	-0.05
# 17	1.15	1.12	1.14
# 18	1.24	1.11	1.13

Supplementary Table S3. Standardized individuals Z scores obtained by participants assessed with the PPT, reported separately for the right dominant hand, left hand and bimanual trials.

	Pardue Pegboard Test		MFT frequency		Minnesota Dexterity Test	
	M	*ES*	*M*	*ES*	*M*	*ES*
Novice	14	-0.10	170	0.50	192 s	1.82
Intermediate	19	2.11	182	1.10	178 s	2.54
Expert	23	4.13	211	2.56	154 s	3.32

Supplementary Table S4. Mean scores and Equivalent scores calculated for each test used to assess hand motor ability of the three actors performing the object manipulation displayed in the videos. Abbreviations: M, Mean Score; ES, Equivalent Score; MFT, Maximum Finger Tapping.

Subject	Left SPL coordinates	Left PMd coordinates	Left IPS coordinates	Left PMv coordinates
	ROIs max overlap -28 -56 58	ROIs max overlap -26 -8 54	ROIs max overlap -36 -40 48	ROIs max overlap -54 8 30
1	-28 -56 58	-26 -10 56	-32 -42 46	-56 6 32
2	-26 -54 56	-28 -10 56	-34 -36 46	-54 8 32
3	-30 -52 60	-28 -6 58	-36 -40 44	-52 10 30
4	-26 -54 58	-28 -10 52	-36 -38 44	-50 6 34
5	-30 -58 58	-26 -4 56	-38 -40 46	-56 10 32
6	-30 -58 60	-26 -10 52	-34 -38 46	-54 6 34
7	-32 -56 60	-28 -10 54	-38 -40 46	-52 6 32
8	-28 -58 56	-28 -10 50	-36 -42 44	-56 8 28
9	-32 -54 62	-28 -8 56	-32 -36 50	-54 12 30
10	-26 -58 60	-24 -10 54	-36 -40 48	-54 4 34
11	-26 -56 60	-30 -10 54	-36 -38 50	-54 4 32
12	-26 -58 62	-28 -10 58	-38 -44 48	-54 6 32
13	-30 -58 62	-28 -8 58	-40 -38 46	-56 10 28
14	-26 -60 58	-28 -8 52	-38 -42 46	-54 8 30
15	-28 -52 62	-26 -8 52	-34 -40 48	-56 10 26
16	-30 -56 62	-30 -10 52	-32 -40 50	-50 6 32
17	-30 -54 60	-26 -10 50	-34 -38 48	-56 4 34
18	-28 -58 62	-26 -8 56	-34 -40 48	-56 6 34

Supplementary Table S5. MNI location of each subject's peak voxels in the left SPL, PMd, IPS and PMv ROIs (Fig. 5A).

Multimedia Legends

Supplementary Video 1. Video clip representing an example of action presented to participants during fMRI, showing a coin manipulation performed by the naïve model (AO Novice).

Supplementary Video 2. Video clip representing an example of action presented to participants during fMRI, showing the naïve model (AO Novice) manipulating a small ball.

Supplementary Video 3. Video clip representing an example of action presented to participants during fMRI, showing a coin manipulation performed by a model with intermediate level of hand motor skills, after 2-month training (AO Intermediate).

Supplementary Video 4. Video clip representing an example of action presented to participants during fMRI, showing the model with intermediate level of hand motor skills manipulating a small ball (AO Intermediate).

Supplementary Video 5. Video clip representing an example of action presented to participants during fMRI, showing a coin manipulation performed by the expert model (professional juggler) (AO Expert).

Supplementary Video 6. Video clip representing an example of action presented to participants during fMRI, showing the expert model (professional juggler) (AO Expert) manipulating a small ball.

References

S1. Bobick, A. F. Movement, activity and action: The role of knowledge in the perception of motion. *Philos. Trans. R. Soc. Lond. B Biol. Sci.* **352**, 1257–1265 (1997).

S2. Schippers, M. B., Roebroeck, A., Renken, R., Nanetti, L. & Keysers, C. Mapping the information flow from one brain to another during gestural communication. *Proc. Natl. Acad. Sci. (USA)* **107**, 9388-9393 (2010).

S3. Cross, E.S., Liepelt, R., Hamilton, A.F., Parkinson, J., Ramsey, R., Stadler, R. & Prinz, W. Robotic movement preferentially engages the action observation network. *Hum. Brain. Mapp.* **33**, 2238-2254 (2012).

S4. Friston, K. J., Holmes, A. P., Price, C. J., Büchel, C. & Worsley, K. J. Multisubject fMRI studies and conjunction analyses. *Neuroimage* **10**, 385-396 (1999).

S5. Friston, K. J., Glaser, D. E., Henson, R. N., Kiebel, S., Phillips, C. & Ashburner, J. Classical and Bayesian inference in neuroimaging: applications. *Neuroimage* **16**, 484-512 (2002).
